# Concurrent omphalomesenteric duct cyst and ileal diverticulum causing small bowel obstruction; a case report

**DOI:** 10.1016/j.ijscr.2022.107004

**Published:** 2022-03-30

**Authors:** Hooman Bahrami-Motlagh, Maryam Sadeghi, Maryam Amerifar, Shahram Sabeti, Seyed Parviz Rezaee, Hassan Peyvandi

**Affiliations:** a Department of Radiology, Loghman-Hakim Hospital, Shahid Beheshti University of Medical Sciences, Tehran, Iran; b Department of Pathology, Loghman-Hakim Hospital, Shahid Beheshti University of Medical Sciences, Tehran, Iran; c Department of Surgery, Loghman-Hakim Hospital, Shahid Beheshti University of Medical Sciences, Tehran, Iran

**Keywords:** OMD, omphalomesenteric duct, Omphalomesenteric duct, Meckel's diverticulum, Omphalomesenteric cyst, Bowel obstruction, Case report

## Abstract

**Introduction and importance:**

The omphalomesenteric duct (OMD) usually involutes by the ninth gestational week. If this obliteration fails, OMD remnant will result in different pathologies mostly in the pediatrics and infrequently in adults. The most well-known OMD remnant disease is Meckel's diverticulum. Omphalomesenteric cyst is rather rare, and their combination is even more exceptional with few cases in literature.

**Case presentation:**

We present an adolescent patient with nausea and vomiting and occasional periumbilical abdominal pain who was diagnosed with concurrent omphalomesenteric cyst and ileal diverticulum, causing internal hernia and bowel obstruction that underwent surgery.

**Clinical discussion:**

OMD remnants mostly present in childhood with symptoms of intestinal obstruction, and rarely internal hernias for which conservative management is usually not curative, warranting surgery. Imaging presence of cystic lesion in mid abdomen in young patient with bowel obstruction should raise the suspicion for OMD remnants. Presence of OMD cyst together with Meckel's diverticulum necessitates more extensive resection, rare concurrence which is better to be prepared for in advance.

**Conclusion:**

Preoperative radiologic workup is helpful to diagnose the obstruction and its probable cause. Presence of periumbilical cyst should raise the suspicion of OMD remnant specially in young adults with previous episodes of crampy abdominal pain and obstruction without history of abdominal surgery. Being familiar with possible concurrence of OMD cyst and Meckel's diverticulum will increase preparedness at the time of surgery.

## Highlights

• The most well-known OMD remnant disease is Meckel's diverticulum.
• Concurrent omphalomesenteric cyst and ileal diverticulum is rare.
• Preoperative radiologic workup is helpful to diagnose the obstruction and its cause.
• Presence of periumbilical cyst should raise the suspicion of OMD remnant.
• Concurrent OMD cyst with diverticulum might warrant surgical intervention.
• Familiarity with OMD cyst & diverticulum concurrence grows preparedness at surgery.

## 1 Background

The omphalomesenteric duct (OMD) is a structural connection between the primitive gut and the yolk sac. Failure of obliteration of OMD may result in various anomalies, with Meckel's diverticulum being the most common. Other less common abnormalities include OMD cysts, fistula and fibrous band between the ileum and umbilicus [1], [2].

Abnormal obliteration of OMD is classified into three types. Type 1 is when the vitelline duct remains patent on both sides, resulting in umbilical discharge. Type 2 is when either end of the duct is patent, with the patency of the distal portion resulting in OMD fistula and the patency of the proximal portion resulting in Meckel's diverticulum. Type 3 is when the mid portion of OMD is patent with atresia of both ends, also known as OMD cysts [3], [4]. These remnants may lead to rare complications such as herniation and bowel obstruction [5].

Here, we present a rare case of concomitant omphalomesenteric cyst and Meckel's diverticulum in an adolescent, resulting in bowel obstruction.

This work has been reported in line with the SCARE criteria. [6]

## 2 Case presentation

A 15-year-old male presented to our hospital in January 2021 with colicky abdominal pain mostly centered in peri-umbilical area. It had started the night before admission and was later accompanied by loss of appetite, nausea and vomiting.

He mentioned previous episodes of nausea and vomiting with occasional periumbilical abdominal pain in childhood which was managed conservatively and was not referred for sub-special medical evaluation.

He denied any history of diarrhea, constipation or fever. He had no previous gastrointestinal surgery, underlying medical condition or any allergies. His previous drug history was limited to oral anti-emetics for nausea. He had no family history of intestinal or any sort of genetic disorders.

Physical examination revealed moderate abdominal distention and periumbilical tenderness without rebound tenderness. Routine laboratory tests were normal.

**Fig. 1:**
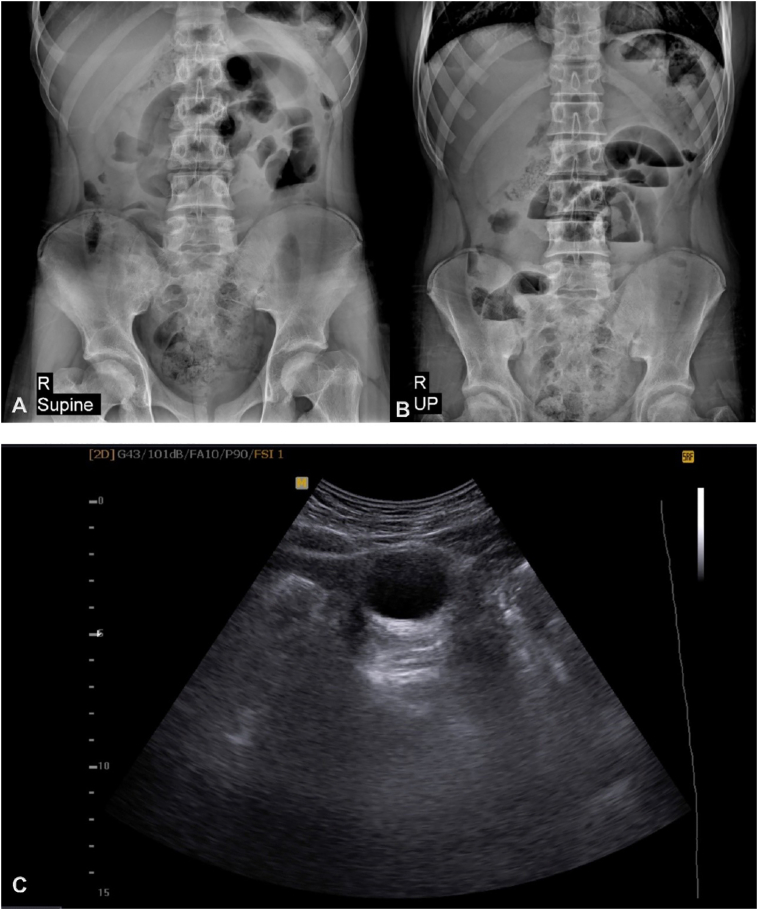
Abdominal X-rays, supine (A) and upright (B), demonstrate distended small bowel loops in mid-abdomen with air-fluid levels in favor of small bowel obstruction. Ultrasound image (C) shows a cystic structure underneath the abdominal wall.

In supine and upright abdominal x-ray, there were several distended small bowel loops with air-fluid levels indicating small bowel obstruction (Fig. 1A, B).

Abdominal ultrasound was performed and revealed a cystic lesion in mid-abdomen close to the undersurface of umbilicus (Fig. 1C). No definite communication with umbilicus or bladder could be identified. There were several dilated bowel loops in the vicinity and small volume of free fluid in pelvic cavity.

**Fig. 2:**
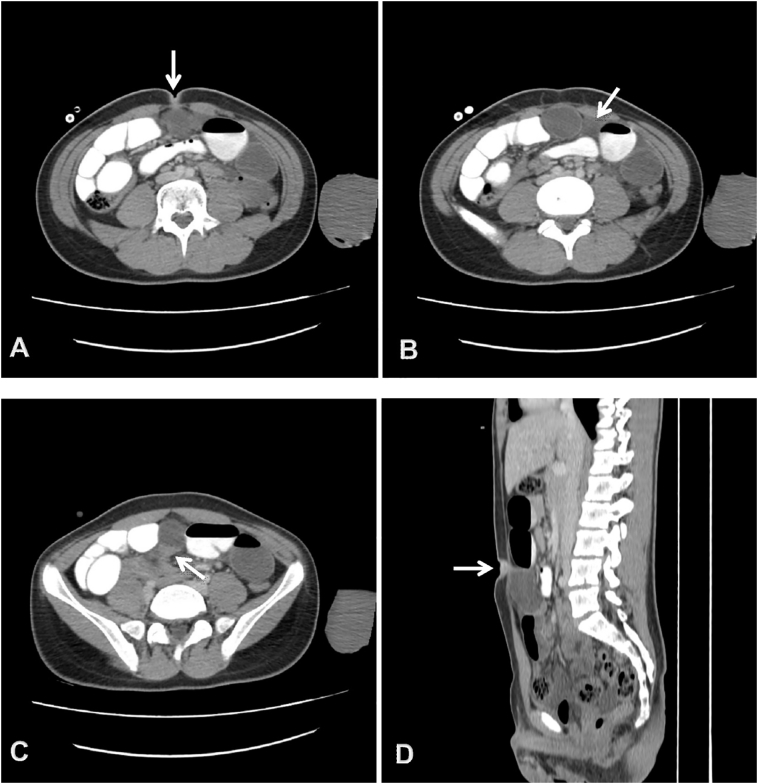
Abdominal CT scan: three sequential axial images from top to bottom (A-C) and sagittal reconstruction (D) demonstrate a midline cyst. The superior aspect seems to be attached to the umbilicus (arrow in A, D) suggestive for omphalomesenteric remnant and the inferior portion is in contact with small bowel loops (arrow in C) depicting mechanical obstruction. Dilated proximal small bowel loops are opacified. Small volume of interloop fluid is present (arrow in B).

Abdominopelvic computed tomography with contrast was performed. In mid-abdomen underneath the anterior wall, there was a cyst measuring 63 ∗ 39 mm. The upper portion of the cyst was in contact with the umbilicus suggestive for omphalomesenteric remnant. Proximal small bowels were distended with abrupt transition along the lower aspect of the cyst in favor of mechanical obstruction related to mentioned cyst. Mild interloop and pelvic free fluid identified, accompanied with several non-significant mesenteric lymph nodes (Fig. 2).

**Fig. 3:**
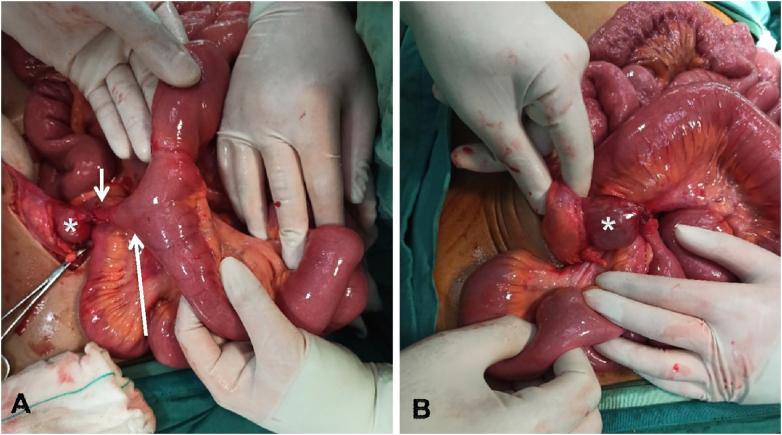
Pictures from laparotomy demonstrating (A) the cyst (asterisk) attached to anterior abdominal wall with a fibrotic band (short arrow) and to the Meckel's diverticulum (long arrow). Small bowel loops were rotated over the fibrotic band, leading to mechanical obstruction (B).

Patient underwent abdominal laparotomy in our general hospital by one of our associate professors of general surgery and their senior trainee with 3 years of surgical specialty training. A midline cyst was discovered, attached via a fibrotic band to the anterior abdominal wall in the umbilical region. The other end was connected with an ileal diverticulum (Fig. 3A). Small bowel loops were herniated internally around the lesion, causing high grade small bowel obstruction (Fig. 3B) Moderate upstream small bowel distention was present without evidence of acute ischemia. The fibrotic band, cyst and accompanying diverticulum were excised from the abdominal wall and resection site at small intestine and abdominal wall were repaired. Small bowel milking was performed and ileocecal valve was proven to be patent. Other parts of the gastrointestinal tract appeared unremarkable.

The patient recovered without complication and was discharged after two days.

**Fig. 4:**
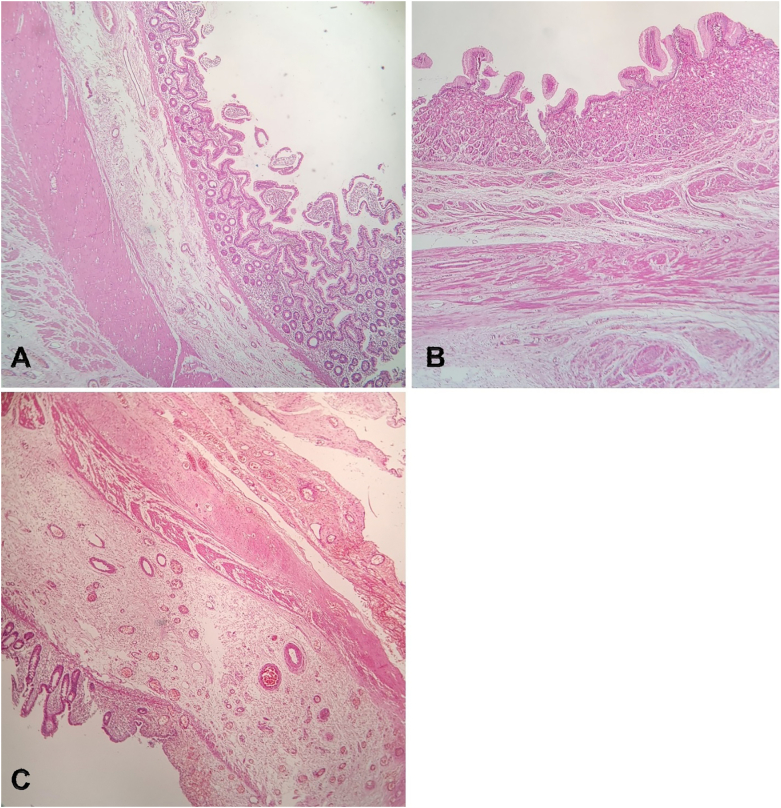
Microscopic examination showing cyst wall lined by small intestinal mucosa (A), gastric mucosa (B) and depicting transmural ulceration (C).

Gross pathologic evaluation of the resection specimen showed omphalomesenteric duct cyst measuring 9 ∗ 5 ∗ 4 cm, attached to a fibrotic band measuring 3 cm in length and 0.5 cm in diameter and continued as Meckel's diverticulum, measuring 1 cm in length and 2 cm in diameter. Histopathologic evaluation of the sample showed small intestinal wall (Fig. 4A) with gastric ectopia (Fig. 4B) accompanied with transmural ulceration (Fig. 4C). No evidence of malignancy noted in the sample.

## 3 Discussion and conclusion

The omphalomesenteric duct usually involutes by the ninth gestational week. If this obliteration fails, OMD remains which causes pathologies mostly in the pediatrics and infrequently in adults [7]. These abnormalities occur based on how patent is the remaining duct connecting the ileum and umbilicus [8]. The most well-known OMD remnant disease is Meckel's diverticulum. Omphalomesenteric cyst is rather rare [9], and their combination is even more exceptional and rare cases found in literature with such presentation.

Nayak et al. reported an 8 year old child with concurrent presentation of OMD cyst, fibrous band and Meckel's diverticulum who had persistent abdominal pain for two years and was treated surgically without any complications [10]. Loannidis et al. presented another case of a 23 year old male with two years history of umbilical discharge who was diagnosed with simultaneous presentation of OMD cyst with a sinus tract at one end and a fibrous band on the other end connecting it to Meckel's diverticulum [11].

We encountered small bowel obstruction associated with a periumbilical cyst in our case. These cysts are classified according to the type of embryonic remnants into three subgroups of uracal, omphalomesenteric duct and round ligament of the liver [12].

Urachal disorders are usually diagnosed by detecting their connection with the bladder in cross sectional imaging [12] which was not present in our case. Recanalization of umbilical vein in ligamentum teres can be easily detected by Doppler ultrasound [13]. CT scan is also helpful in depicting patent umbilical vein together with other findings of portal hypertension [12]. Both anomalies were excluded in our patient according to imaging findings and OMD remnant was suggested as the most probable preoperative diagnosis.

OMD remnants mostly present in childhood [14] causing symptoms like intestinal obstruction, abdominal pain and internal hernias [15] although the latter is very rare [16]. If they become symptomatic they should be treated via surgery and conservative management is usually not curative [17]. Detection of periumbilical cyst in preoperative ultrasound or CT scan in young patient with bowel obstruction should raise the suspicion for OMD remnant as underlying etiology and expedite the intervention. Hypothetically, presence of two structural derangements including both OMD cyst and diverticulum can increase the chance of becoming symptomatic and the need for resection although this should be further investigated in large scale studies. Nevertheless, being familiar with their possible concurrence will increase preparedness at the time of surgery.

In conclusion, we presented a rare case of an adolescent patient with concurrent omphalomesenteric cyst and Meckel's diverticulum, causing internal hernia and bowel obstruction who underwent surgery. Presence of periumbilical cyst in young adult with previous episodes of crampy abdominal pain and obstruction should raise the suspicion of OMD remnants as the underlying etiology while being familiar with the rare concurrence of OMD cyst with diverticulum will increase the preparedness at the time of surgery.

## Availability of data and materials

The datasets used and/or analysed during the current study are available from the corresponding author on reasonable request.

## Sources of funding

Not applicable.

## Ethics approval

Ethics approval and consent have been obtained from ethics committee of Loghman Hakim Hospital.

## Consent

Written informed consent was obtained from the patient and his parents for publication of this case report.

## Authors' contributions

HBM contributed interpretation of radiological data and critical revision of the manuscript.MS contributed acquisition of data and drafting of the manuscript. MA contributed acquisition of data and revision of the manuscript. SS contributed interpretation of pathology data. SPR and HP contributed surgical procedures of this case report. SA contributed the interpretation of radiological data. All authors read and approved the final manuscript and have agreed to be accountable for the authors' own contributions and ensured that questions related to the accuracy or integrity of any part of the work, even ones in which the author was not personally involved, can be appropriately investigated, resolved, and the resolution be documented in the literature.

## Research registration

Not applicable.

## Guarantor

Dr. Hooman Bahrami-Motlagh, Dr. Maryam Sadeghi.

## Declaration of competing interest

The authors declare that they have no conflicts of interests.
